# Phase-stable, multi-µJ femtosecond pulses from a repetition-rate tunable Ti:Sa-oscillator-seeded Yb-fiber amplifier

**DOI:** 10.1007/s00340-016-6611-9

**Published:** 2016-12-20

**Authors:** T. Saule, S. Holzberger, O. De Vries, M. Plötner, J. Limpert, A. Tünnermann, I. Pupeza

**Affiliations:** 1 Max-Planck-Institut für Quantenoptik, Hans-Kopfermann-Str. 1, 85748 Garching, Germany; 2 Ludwig-Maximilians-Universität München, Am Coulombwall 1, 85748 Garching, Germany; 3 Fraunhofer Institute for Applied Optics and Precision Engineering, Albert-Einstein-Str. 7, 07745 Jena, Germany; 4 Institut für Angewandte Physik, Friedrich-Schiller-Universität Jena, Alber-Einstein-Str. 15, 07745 Jena, Germany; 5 Helmholtz-Institut Jena, Fröbelstieg 3, 07743 Jena, Germany; 6 Active Fiber Systems GmbH, Wildenbruchstr. 15, 07745 Jena, Germany; 7 Present Address: Menlo Systems GmbH, Am Klopferspitz 19a, 82152 Martinsried, Germany

**Keywords:** Phase Noise, Frequency Comb, Master Oscillator Power Amplifier, Attosecond Pulse, Phase Jitter

## Abstract

We present a high-power, MHz-repetition-rate, phase-stable femtosecond laser system based on a phase-stabilized Ti:Sa oscillator and a multi-stage Yb-fiber chirped-pulse power amplifier. A 10-nm band around 1030 nm is split from the 7-fs oscillator output and serves as the seed for subsequent amplification by 54 dB to 80 W of average power. The µJ-level output is spectrally broadened in a solid-core fiber and compressed to ~30 fs with chirped mirrors. A pulse picker prior to power amplification allows for decreasing the repetition rate from 74 MHz by a factor of up to 4 without affecting the pulse parameters. To compensate for phase jitter added by the amplifier to the feed-forward phase-stabilized seeding pulses, a self-referencing feed-back loop is implemented at the system output. An integrated out-of-loop phase noise of less than 100 mrad was measured in the band from 0.4 Hz to 400 kHz, which to the best of our knowledge corresponds to the highest phase stability ever demonstrated for high-power, multi-MHz-repetition-rate ultrafast lasers. This system will enable experiments in attosecond physics at unprecedented repetition rates, it offers ideal prerequisites for the generation and field-resolved electro-optical sampling of high-power, broadband infrared pulses, and it is suitable for phase-stable white light generation.

## Introduction

Around the turn of the last century, the generation of visible/near-infrared few-cycle pulses became a matter of course in laser laboratories. The ability to control the electric field of such pulses enabled ground-breaking applications like high-precision frequency comb spectroscopy [1] and led to the establishment of new research fields like attosecond science [2, 3]. Titanium-sapphire (Ti:Sa) oscillators can be considered the workhorse in this field, since they are the most widespread technology employed to seed laser systems generating phase-stable few-cycle pulses. Important contributions to the success of these oscillators are their large optical bandwidth, leading to the direct generation of few-cycle pulses, and the excellent phase stability achievable with feed-back [4] and feed-forward stabilization schemes [5]. However, a major drawback of Ti:Sa amplifiers is the high thermal absorption and thermal lensing in the gain medium, which restricts the average powers to a few tens of Watts even with cryogenic cooling [6], thus limiting high-pulse-energy operation to repetition rates significantly lower than 1 MHz. Recently, Yb-based laser technology has rapidly progressed as a powerful competitor to the well-established Ti:Sa technology. The superior thermal properties of Yb-doped active media enable an improvement of several orders of magnitude in terms of average power [7–11]. To overcome the disadvantage of a significantly narrower gain bandwidth, several post-amplification nonlinear pulse compression techniques have been developed [9–14]. Recently, from an Yb:YAG thin-disk oscillator followed by two nonlinear compression stages, pulses of 2.2 cycles with 6 W of average power at a repetition rate of 38 MHz and with a phase jitter of 270 mrad (out-of-loop phase noise integrated in the band between 1 Hz and 500 kHz) were demonstrated [10].

In this study, we combine a feed-forward stabilized Ti:Sa master oscillator with an Yb-fiber power amplifier, resulting in a high-power, ultrashort-pulse laser system with several unique advantages for electric-field-resolved metrology. First, the high phase stability achieved with the Ti:Sa frontend is largely preserved upon amplification of a 10-nm band around 1030 nm by about 54 dB to 80 W. Additional phase fluctuations introduced by the multi-stage, chirped-pulse amplifier (CPA) and by subsequent nonlinear compression to about 30 fs were compensated for by a feed-back loop, resulting in an unprecedentedly small overall phase jitter of the high-power pulse train of less than 100 mrad (out-of-loop phase noise, integrated in the band between 0.4 Hz and 400 kHz). Due to its phase stability, this source is particularly well suited to drive cavity-enhanced high-order harmonic generation (HHG) for the generation of extreme ultraviolet (XUV) frequency combs [15, 16] and of XUV attosecond pulses [16–18]. Second, the master oscillator power amplifier (MOPA) approach readily enables the use of a high-frequency pulse picker after the low-power oscillator [19], allowing for a tunable repetition frequency (18.5, 24.7, 37 and 74 MHz). And third, the Ti:Sa oscillator produces a 74-MHz train of 7-fs pulses, inherently synchronized with the pulses amplified in the Yb-fiber CPA. These could be used for instance as a pump in time-resolved photoelectron emission microscopy [20], where attosecond XUV probe pulses are generated with the CPA output. Another application could be electro-optical sampling of broadband infrared radiation [21] generated with the CPA output, where a repetition-rate ratio of a factor of 2 between the sampling pulses and the long-wavelength field enables ultralow-noise lock-in detection [22].

## Experimental setup

**Fig. 1:**
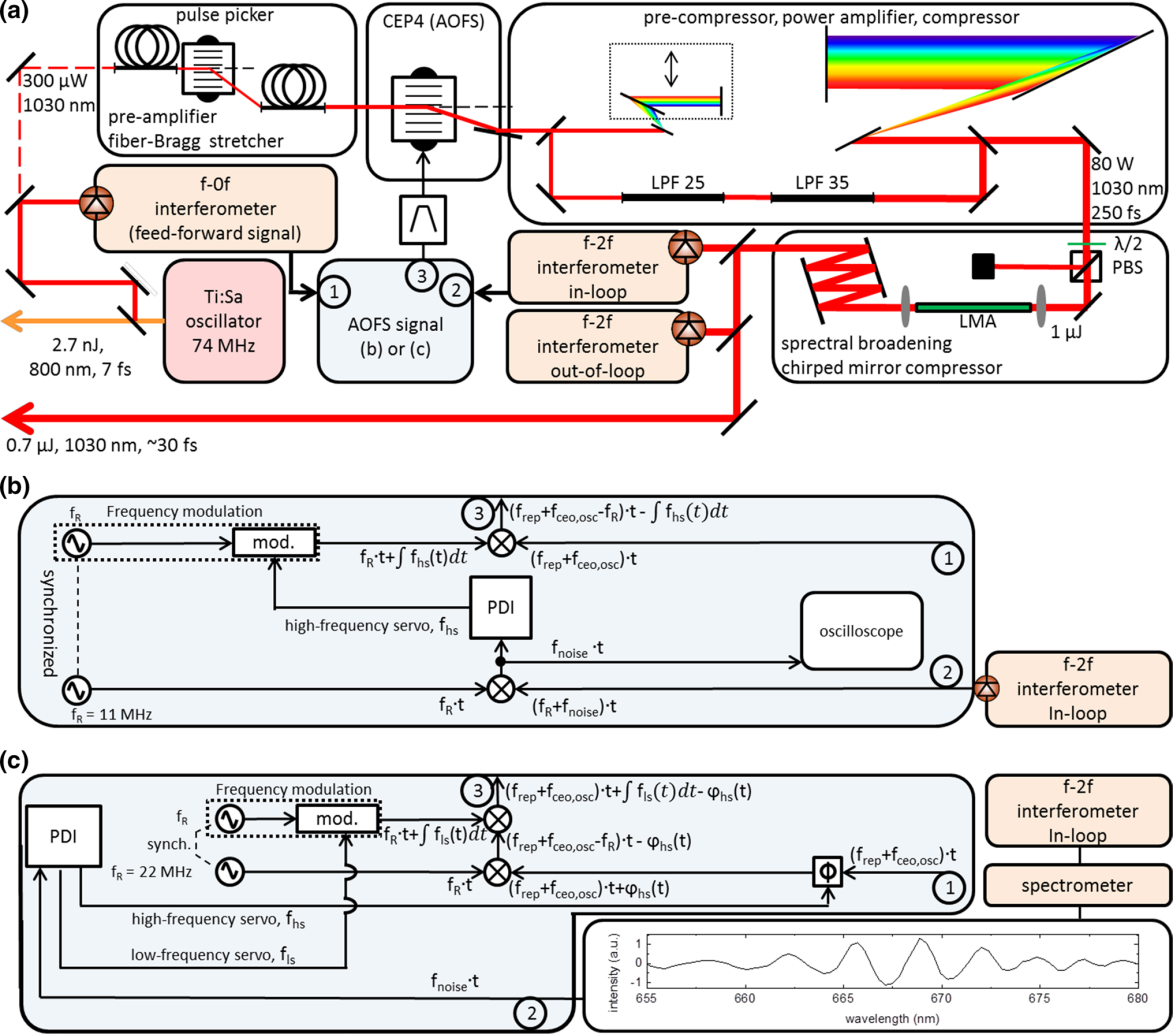
**a** Schematic of the experimental setup. A 300-µW, 10-nm band around 1030 nm of the Ti:Sa output is split from the main beam and serves as the seed for the Yb-fiber amplifier. After pre-amplification, reduction of the repetition rate and stretching to around 200 ps [19], the pulse train is stabilized to a pre-chosen carrier-envelope-offset frequency *f*_ceo_ by a feed-forward scheme using an AOFS (see text for details) using a CEP4 stabilization unit (Spectra-Physics, Femtolasers). The main amplification to 80 W is accomplished by two amplification steps in Yb-doped large-pitch fibers (LPF) [23] and two grating compressors. Subsequently, a rod-type LMA fiber and chirped mirrors are used to generate 0.7-µJ, ~30-fs pulses. The *f*_ceo_ is then measured with a *f* − 2*f* interferometer and stabilized by a feed-back scheme using frequency modulation of the feed-forward AOFS signal by the servo output of a proportional-derivative integral (PDI) lock box. **b** AOFS signal generation for stabilization to *f*_R_ = *f*_ceo_ = 11 MHz. The frequency of the common feed-forward signal *f*_rep_ + *f*_ceo,osc_ (1) is reduced by mixing with a 11-MHz reference signal and then stabilized by frequency modulation. The error signal is derived from a heterodyne detection scheme with the beatnote (2). For characterization, the phase noise is recorded by sampling this signal with an oscilloscope. The phase of the RF signals is indicated along the *black lines*. **c** Stabilization scheme for *f*_ceo_ = 0 MHz. The *f* − 2*f* interferometer is modified to a spectrally resolved configuration. The stabilization is achieved by first decreasing the AOFS carrier frequency by *f*_R_ = 22 MHz and afterwards increasing it by the same synchronized frequency with a small modulation derived from the interferogram (2), containing the feed-back signal. For stability reasons, a phase shifter (*Φ*) was also applied in a faster servo loop in order to fine-tune the lock. The *inset* shows an interferogram with subtracted background

The experimental setup is shown in Fig. 1a. The Ti:Sa frontend provides 7-fs pulses with 200 mW of average power at 74-MHz repetition rate. The 10-nm band around 1030 nm carries 300 µW of average power and acts as the seed for the Yb-fiber amplifier. In a first step, the repetition rate is (optionally) reduced by a fast acousto-optic modulator (AOM) pulse picker, and the resulting pulses are amplified to 150 mW and stretched to around 200 ps by a chirped fiber Bragg grating [19]. To minimize additional intensity and phase noise, the pulse picker is operated in synchronicity with the repetition rate as described in [19]. After phase stabilization by an acousto-optic frequency shifter (AOFS) operated in the feed-forward stabilization scheme [5], the subsequent amplification stages and the compression comprise two Yb-large-pitch-fiber (LPF) amplifiers [23], two grating compressors and a rod-type large-mode-area (LMA) fiber for spectral broadening, followed by a chirped-mirror compressor.

To fine-tune the pulse duration at the CPA output, the pre-compressor is motorized to control the distance of the gratings. It only influences the pulse duration of the stretched pulses by up to 10 ps and allows for stable and precise control of the output pulse duration. The estimated B-integral of the system amounts to around 0.1 rad irrespective of the setting of the pre-compressor. The dispersion of the gratings is matched up to the sixth order.

The overall phase noise at the exit of the system is measured with two independent *f* – 2*f* interferometers [24]. The first interferometer (in-loop) is used to generate a feed-back error signal which is combined with the original feed-forward stabilization by adding a frequency modulation to the RF signal used to drive the AOFS (signal 3 in Fig. 1) and thus also a phase modulation (see phase along the black lines in Fig. 1b, c). The second (out-of-loop) interferometer is used to determine the actual phase noise outside of the locking loop.

The amplification process contains several potential phase noise sources, including amplification noise [25], grating jitter (mechanical and air fluctuations) [26] and residual noise during the pulse picking in the AOM [19]. Furthermore, the mechanical jitter in the interferometers themselves and the supercontinuum generation necessary for *f* − 2*f* detection add to the measured phase noise [27].

**Fig. 2:**
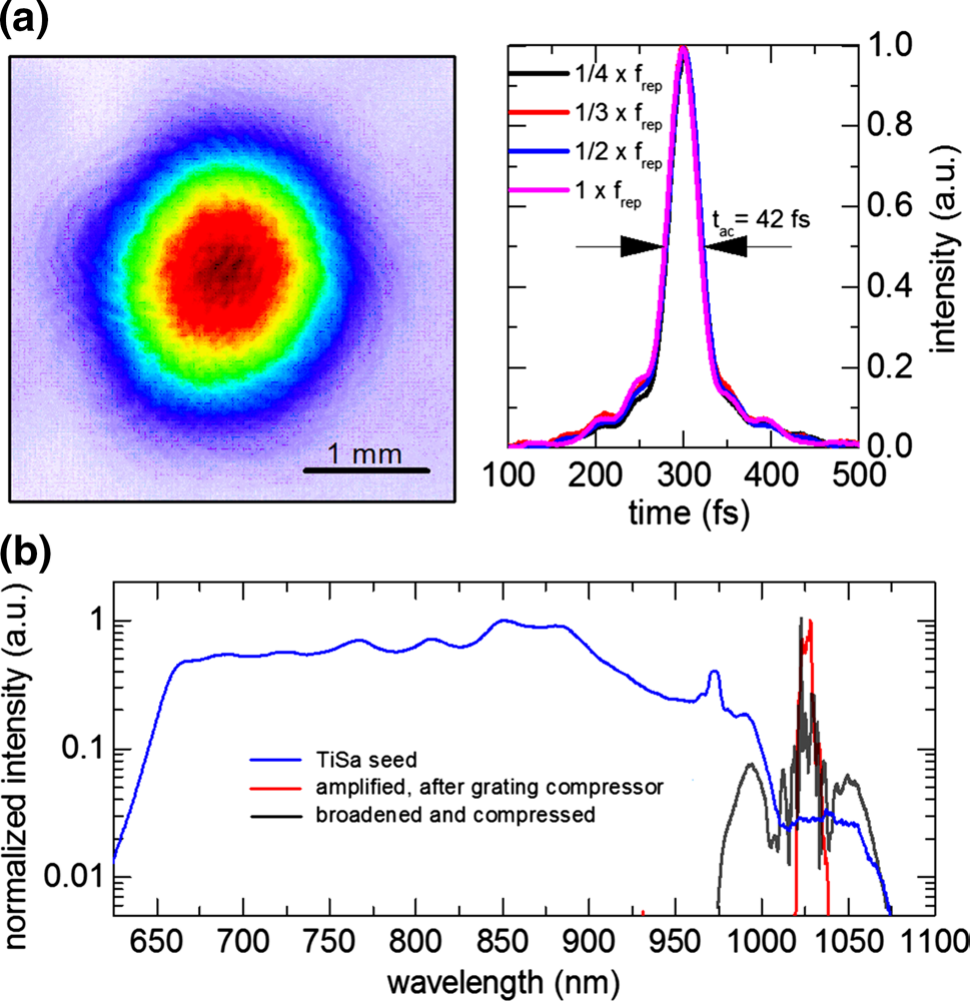
**a** Beam profile and autocorrelation traces at the output of the MOPA system. Different repetition rates show the same autocorrelation trace duration of 42 fs, corresponding to 32 fs for a Gaussian pulse. **b** Spectra of the seed (*blue*), amplified (*red*) and compressed pulses (*black*)

The MOPA system is capable of delivering a 250-fs (all pulse durations are given in full width at half their intensity maximum), 80-W pulse train at repetition rates ranging from 74 MHz down to 18.5 MHz, which corresponds to a maximum of 4.3 µJ. The solid-core-fiber-based spectral broadening employed here is limited to around 1-µJ input pulse energy by self-focusing-induced damage in the fiber. This is, however, not a fundamental limitation in view of the recently demonstrated power scalable compression schemes in bulk [9, 28] as well as in kagome fibers [14]. The beam profile and autocorrelation traces for the four repetition rates are shown in Fig. 2a. The pulse durations, assuming a Gaussian-shaped pulse, are measured to be 32 fs for all considered repetition rates, which is close to the Fourier limit of ~28 fs. The spectra for the seed, amplified and compressed pulses are shown in Fig. 2b.

The value of the carrier-envelope offset frequency *f*_ceo_ of the amplified pulse train can be adjusted by manipulating the signal used for the stabilization in the AOFS, which consists of three contributions, as shown in Fig. 1b, c. Signal 1 is the feed-forward signal consisting of the repetition frequency and the carrier-envelope offset frequency of the oscillator (*f*_rep_ + *f*_ceo,osc_) which, in normal operation, shifts *f*_ceo_ to 0 MHz in the -first diffraction order of the AOFS. The value of *f*_ceo_ can optionally be tuned by mixing an additional frequency with an RF mixer and a signal generator (signal 2). The combined signal is then band-pass filtered, amplified and fed to the AOFS (signal 3).

## Results

### Carrier-envelope-offset frequency of 11 MHz

In a first configuration, we set the driving frequency of the AOFS to *f*_rep_ + *f*_ceo,osc_-11 MHz (a value of 71 MHz, the free running oscillator offset frequency *f*_ceo,osc_ is kept around 8 MHz by means of intracavity wedges and crystal temperature). This is depicted in Fig. 1b. Using this scheme, any value of the carrier-envelope-offset frequency of the output pulse train can be chosen, provided that the AOFS is suited for that frequency.

**Fig. 3:**
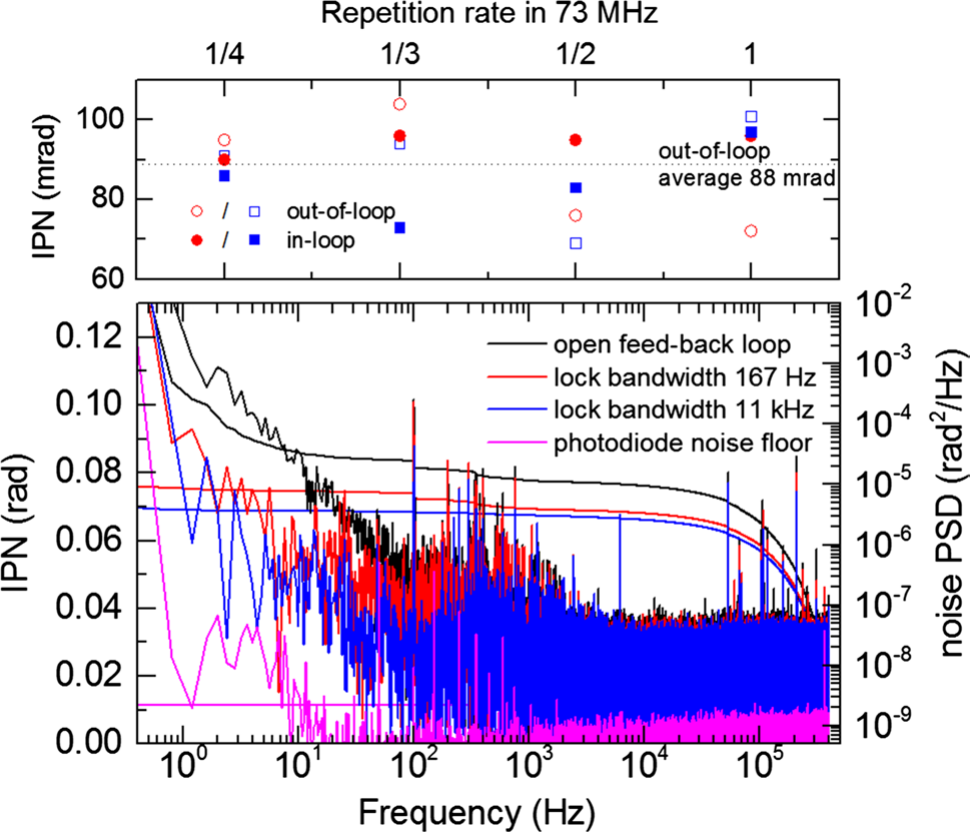
Phase noise measurements (integrated phase noise, IPN, and noise power spectral density, PSD) for measurement times up to 2.5 s and for different pulse repetition rates. *Black line* noise at open feed-back locking loop using only the feed-forward stabilization. *Red and blue* closed-loop operating with a bandwidth of 167 Hz and 11 kHz, respectively. The upper panel summarizes the performance at different repetition rates: 18.5, 24.7, 37 and 74 MHz

**Fig. 4:**
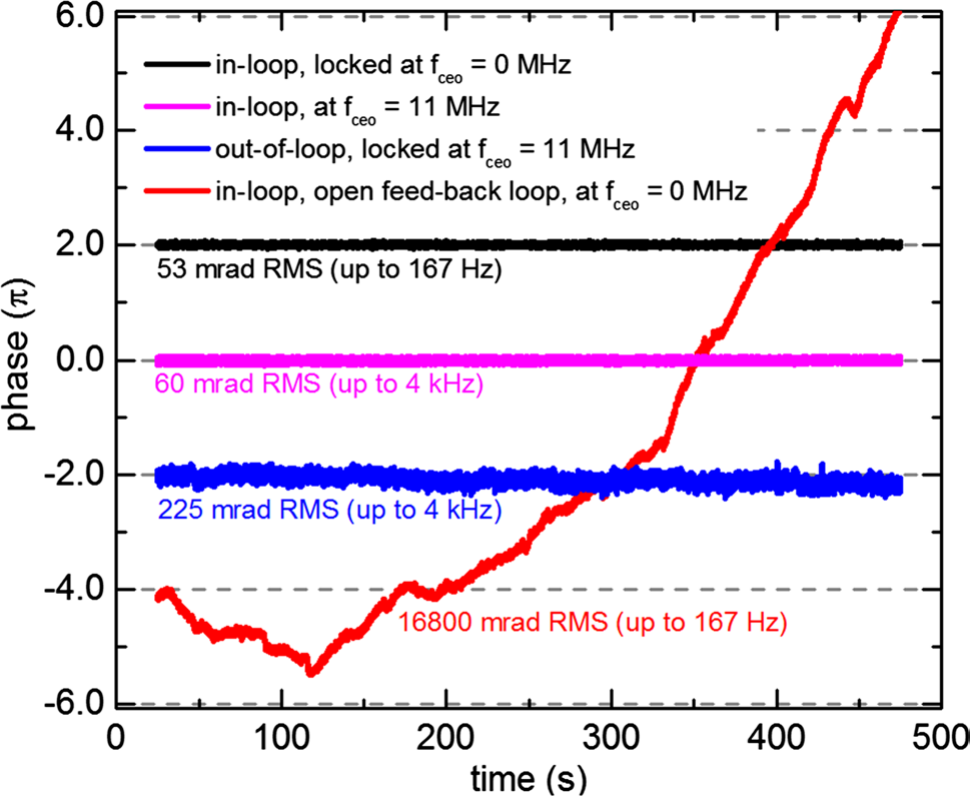
Long-term phase noise measurement at 18.5 MHz repetition rate for *f*_ceo_ = 0 MHz and *f*_ceo_ = 11 MHz. *Black and red* in-loop measurements for the 0-MHz case, the *red* one was taken with an open feed-back loop (i.e., only feed-forward stabilization). *Magenta and blue* in-loop and out-of-loop phase fluctuations when *f*_ceo_ = 11 MHz. The traces are offset for clarity

The feed-back loop is achieved by a heterodyne detection scheme using the detected beatnote and a synchronized RF generator operating at the same frequency as the generator used to shift the feed-forward signal. This provides the error signal for the feed-back servo loop. The output of the proportional-derivative-integral (PDI) controller is used to modulate the AOFS driving frequency. The beatnote used in this work was at least 30 dB above the noise floor at 100 kHz resolution and video bandwidth in order to achieve a tight lock and a low-noise measurement. The results for different repetition rates are shown in Fig. 3. The lower part of the figure depicts a typical out-of-loop measurement including the photodiode noise floor (magenta), the phase noise with disabled feed-back but still enabled feed-forward locking servo (black) and two different closed-loop operation modes with locking bandwidths of 167 Hz (red) and 11 kHz (blue). The MOPA system shows an integrated phase noise (IPN) of 76 mrad and 69 mrad, respectively, at closed-loop operation and 145 mrad at open-loop operation in the frequency range of 0.4 Hz to 400 kHz, corresponding to an improvement by roughly a factor of 2 compared to state-of-the-art phase-stable multi-MHz Yb-based systems [10, 29]. The upper part is a summary of the in-loop and the out-of-loop measurements for different repetition rates (18.5, 24.7, 37 and 74 MHz), showing that within the measurement accuracy of ~10% the IPN is independent on the pulse picking factor. Furthermore, the values for the out-of-loop results are very close to those for the in-loop measurements, showing that the lock does indeed correct for real phase noise and that these are not measurement artifacts, as the lock would add noise in the out-of-loop configuration in such a case. To the best of our knowledge, the average value of (88 ± 7) mrad RMS phase noise integrated between 0.4 Hz and 400 kHz for an out-of-loop measurement is the best value achieved so far with ultrashort high-power multi-MHz-repetition-rate systems. The 7-mrad error is the standard deviation at the repetition rate of 18.5 MHz, taken for several measurements over the course of half an hour, and it mainly depends on the alignment of the interferometers and on changes in the environment. Average open-loop measurements (black trace) yield a phase noise around 2 times higher than the closed-loop operation, demonstrating the benefit of the additional feed-back loop. The 0.4- to 10-Hz fluctuation in the open-loop trace originates from within the laser system, e.g., from small mechanical jitter in the grating compressors and/or air fluctuations. One noise source in the measurement is in the >100 kHz frequency range. Most probably, this originates from intensity fluctuations which are transformed into phase noise during the octave generation in the *f* − 2*f* interferometer as reported in [27]. Another noise source, located at 100 Hz, was nearly completely suppressed by a housing around the interferometers, meaning that the noise originates from air fluctuations and mechanical vibrations in the interferometer and not from the laser system. The long-term stability is shown in Fig. 4, in-loop (magenta) and out-of-loop (blue). The performance over 7.5 min was investigated for different configurations. The values of 60-mrad in-loop and 225-mrad out-of-loop for the long-term IPN are both unparalleled for high-power multi-MHz systems. The real out-of-loop phase noise is even lower as the statistical fluctuations (<1 Hz, stemming from, e.g., beam pointing, delay jitter in the interferometer, air fluctuations, etc.) in the two interferometers add up and increase the value drastically. For comparison, one of the most stable state-of-the-art 10-kHz Ti:Sa systems reaches a 140-mrad out-of-loop long-term stability at an average power of 8 W [30]. For high-power ultrashort-pulse multi-MHz systems, previous out-of-loop measurements were taken on the order of a few seconds only and yielded a value of 270 mrad (out-of-loop jitter integrated in the bandwidth between 1 Hz and 500 kHz) [10]. For uncompressed pulses, 140 mrad (out-of-loop jitter integrated in the bandwidth between 0.4 Hz and 100 kHz) was achieved [29], albeit this measurement was taken before a grating compressor.

### Carrier-envelope-offset frequency of 0 MHz

By adapting the AOFS signal generation and phase detection in the *f* − 2*f* interferometer, the value of the comb offset can also be set to 0 MHz, as shown in Fig. 1c. The main difference in the *f* − 2*f* interferometer setup is the spectrally resolved detection and the signal processing. The interferometer delivers an interferogram which is digitally evaluated and fed to a PDI controller. The feed-back loop consists of two stages, a faster loop using a 360° phase shifter [30] and a slower one to compensate for drifts over several radians (see red trace in Fig. 4). The slow drift compensation is accomplished by decreasing the AOFS frequency by 22 MHz and afterwards increasing it by the same synchronized frequency with a small additional modulation, corresponding to the feed-back signal. Due to the digital evaluation of the interferogram, the lock was limited to a bandwidth of 167 Hz. The previous measurements at 11-MHz offset frequency, however, showed that the bandwidth of 167 Hz is sufficient as there was no major difference when using a locking bandwidth of 11 kHz (Fig. 3). The results for the long-term measurements are shown in Fig. 4. As no second spectrally resolved 0-MHz *f* − 2*f* interferometer was available, only in-loop measurements can be shown. However, the 11-MHz measurements corroborate that the out-of-loop performance should be on the same order. The necessity of a slow drift compensation for more than 2 rad presents itself in the open-loop trace (red) as the phase drift is larger than 30 rad during 7.5 min. This drift is not possible to lock with only a single 360° phase shifter as demonstrated in [30]. The in-loop closed-loop measurement is shown in black and manifests an extraordinary value of 53 mrad within a bandwidth of 167 Hz which is consistent with the in-loop value of 60 mrad measured at *f*_ceo_ = 11 MHz (magenta trace, bandwidth 4 kHz). This operation is stable over tens of minutes.

## Conclusion and outlook

In conclusion, we have demonstrated an Yb-fiber MOPA system seeded by a Ti:Sa oscillator, achieving excellent phase stability. The repetition rate of the 0.7-µJ, ~32-fs output pulses can be reduced from 74 MHz (fundamental repetition rate of the oscillator) by an integer factor of up to 4 without affecting the pulse parameters. In principle, the repetition rate can be further reduced at the same average power by stronger pulse stretching in the CPA [31] and the pulse duration can be further compressed with alternative, pulse-energy-scalable schemes [9–14]. For all repetition rates, an integrated phase noise of 60-mrad in-loop and 225-mrad out-of-loop was measured in a bandwidth from 2 mHz to 4 kHz. These results have a threefold significance for ultrafast laser technology. First, they demonstrate that the residual phase noise introduced by Yb-fiber amplifiers (including CPA), even for an amplification factor of > 50 dB, is low enough to be readily compensated by a simple feed-back scheme. On the other hand, they indicate that for a high phase stability, a feed-back loop is necessary for the compensation of these phase fluctuations. Second, these results prove that feed-forward-stabilized Ti:Sa oscillators are well suited as low-phase-noise seeders for Yb:fiber systems. Third, they show that a fast AOM pulse picker after the low-power frontend mostly preserves the phase stability irrespective of the picking factor.

The laser system demonstrated here opens up new opportunities in several fields. For instance, the zero-offset-frequency pulse train can be used to drive HHG in a suitable femtosecond enhancement cavity [16, 17] for the generation of attosecond pulse trains and, ultimately, for the generation of isolated attosecond pulses at multi-MHz repetition rates [18]. This will dramatically decrease the measurement times in photoelectron emission microscopy and spectroscopy, in particular allowing for the study of plasmonic fields with a unique combination of nm-scale spatial resolution with sub-femtosecond temporal resolution [20]. Furthermore, the adjustable repetition rate allows for direct studies of cumulative effects such as those observed in HHG in gases at high repetition rates [16]. Another application which will tremendously profit from this MOPA is field-resolved detection of broadband infrared pulses [21], employing the 7-fs pulses generated by the Ti:Sa oscillator for electro-optical sampling with a lock-in detection at half the fundamental oscillator repetition rate [22]. Furthermore, the phase-stable output could be employed as a frequency comb or to drive the generation of ultrabroadband supercontinua, which can be used to obtain high-energy field-synthesized femtosecond transients [32].
